# Generation and characterization of human induced pluripotent stem cells from neuropathologically confirmed multiple system atrophy patient-derived fibroblasts

**DOI:** 10.3389/fimmu.2026.1641981

**Published:** 2026-02-23

**Authors:** Mireia Alemany-Ribes, Alexandra Pérez-Soriano, Almudena Santos, Jacqueline Severino, Adrià Dangla-Valls, Martín Gigirey-Suárez, Dory Cohen, Mario Ezquerra, Ruben Fernandez-Santiago, Manel Fernandez, Maria J. Martí, Veerle Baekelandt, Wouter Peelaerts, Ronald Melki, Yaroslau Compta, Laura Batlle-Morera

**Affiliations:** 1 Centre for Genomic Regulation (CRG), The Barcelona Institute of Science and Technology, Catalonia, Spain; 2 Parkinson’s Disease & Movement Disorders Unit and Lab of Parkinson & Other Neurodegenerative Movement Disorders, Hospital Clínic/IDIBAPS/CIBERNED/European Reference Network for Rare Neurological Diseases (ERN-RND)/Institut de Neurociències "UBNeuro", University of Barcelona, Catalonia, Spain; 3 Institut François Jacob (MIRCen), CEA, and Laboratory of Neurodegenerative Diseases, CNRS, Fontenay-aux-Roses, France; 4 Laboratory for Neurobiology and Gene Therapy, Department of Neurosciences, Leuven Brain Institute, KU Leuven, Leuven, Belgium; 5 Laboratory for Advanced Disease Models, Targeted Drug Development and Gene Therapy (ADVANTAGE), Department of Pharmaceutical Sciences, KU Leuven, Leuven, Belgium

**Keywords:** disease modeling, human induced pluripotent stem cells, multiple system atrophy, oligodendrocytes, quality control, reprogramming

## Abstract

**Background:**

Multiple system atrophy (MSA) is an adult-onset, fatal neurodegenerative disease, classified as a synucleinopathy along with Parkinson’s disease and dementia with Lewy bodies. The etiology of MSA is unknown and the treatment remains symptomatic with limited efficacy. To date most studies have been conducted on genetic mice models, engineered to overexpress α-synuclein (aSyn); and transmission mice models, treated with intracerebral injections of MSA brain extracts or fibrillar aSyn which can be assembled *de novo* or using MSA seeds. An *in vitro* human model is urgently needed to identify the human-specific and even patient-specific molecular mechanisms underlying the disease.

**Method:**

For this purpose, we report the generation of six fully characterized induced pluripotent stem cells (iPSCs) lines, which were reprogrammed from disease-derived skin fibroblasts and represent the two major clinical MSA variants with either predominant parkinsonism (MSA-P) or cerebellar dysfunction (MSA-C). Five of these cases received postmortem neuropathological confirmation of definite MSA diagnosis.

**Results:**

The generated iPSC clones meet the International Society for Stem Cell Research (ISSCR) quality standards for genomic stability and functional pluripotency to ensure experimental reproducibility. None of the clones acquired genetic abnormalities after the reprogramming and the extended culture passage, as evaluated by G-banded karyotyping and digital PCR targeting recurrent iPSC copy number variant hotspots. All the clones expressed pluripotent markers as NANOG, LIN28A and SSEA4 and differentiated efficiently into each of the three embryonic germ layers. Furthermore, we assessed their potential for disease modeling by generating oligodendrocytes (OLs), since the pathologic hallmark of MSA is the aberrant accumulation of aSyn within OLs cytoplasm. We obtained both O4+ oligodendrocyte progenitor cells and MBP+ mature oligodendrocyte cells.

**Conclusion:**

We have created a robust and valuable iPSC collection aimed at correlating the *in vivo* confirmed clinical and pathological variants with the *in vitro* phenotypes. This unique tool can contribute to the understanding of MSA pathophysiology, the discovery of therapeutic targets, and the development of therapeutic screening approaches.

## 1 Introduction

Multiple system atrophy (MSA) is a rare adult-onset neurodegenerative synucleinopathy of unknown etiology. The disease is characterized by autonomic dysfunction and motor symptoms presented by two primary clinical and pathological forms, which are distinguished by either cerebellar symptoms (MSA-C) or parkinsonism with poor response to levodopa (MSA-P) (1). Pathologically, MSA features mostly oligodendroglial α-synuclein (aSyn) aggregates forming glial cytoplasmic inclusions (GCIs), in contrast to Parkinson’s disease, where aSyn predominantly accumulates within neurons. These GCIs are associated with neuronal loss and gliosis, that can result in both olivopontocerebellar atrophy (OPCA) and striatonigral degeneration (SND) (2). Typically, OPCA correlates with MSA-C and SND with MSA-P, but in advanced disease stages both OPCA and SND occur in varying degrees (3). The disease is highly debilitating and affects adults in their mid-fifties and has an overall survival of 9 years (4). Over 50% of people with MSA are usually dependent for daily activities after 5–6 years since disease onset (5). There is no effective treatment for the disease, possibly due to research difficulties, including inaccessibility to disease-specific tissue (only available postmortem), lack of insights into causative genetic or environmental factors, and the absence of reliable disease models to explore new therapeutic strategies (6).

The molecular pathogenesis of the disease, particularly the accumulation of αSyn in oligodendrocytes, remains uncertain. While there is little evidence of clear causative genes, reports of familial cases of the disease suggest potential genetic susceptibility in certain instances (7, 8). Research has focused on exploring the aSyn gene (*SNCA*), through various case-control and genome-wide association studies. While some studies suggest a genetic association between *SNCA* variants and MSA, larger studies have not consistently replicated these findings, and instead have identified variants in genes such as *MAPT*, *APOE*, *COQ2*, *LRRK2*, *SLC1A4*, *SQSTM1*, *EIF4EBP1*, and *GBA* as potential contributors to increased MSA susceptibility (9). These results are often inconsistent and specific links to polymorphisms and monogenic mutations are rare (10). Thus, at present, it is not possible to engineer human cells using genome editing to introduce disease-causing mutations and create genetically controlled models for MSA. In addition, MSA animal models have been generated by overexpressing aSyn (6); however, studies assessing *SNCA* mRNA levels in human brain have not found a clear overexpression pattern (11). Induced pluripotent stem cell (iPSC) technology emerges as a valuable tool for modeling MSA *in vitro*, since it allows us to reprogram somatic cells from patients into an embryonic stem cell-like state followed by differentiation into disease-relevant cell types, better mimicking human pathophysiology (12–14).

Three other groups have previously reported the generation of MSA patients-derived iPSC. Specifically, Nakamoto et al. established two MSA iPSC lines, both belonging to the clinically diagnosed cerebellar subtype, and one carrying compound heterozygous *COQ2* mutations (p.[R387*]/[V393A]), which have been associated to familial MSA without consensus (15). Compagnoni et al. obtained four MSA iPSC lines, two cerebellar and two parkinsonian subtypes, and significantly they also counted with the healthy monozygotic twin of one of the MSA-C patients. The beforementioned iPSCs were subsequently differentiated into neurons to study their mitochondrial respiration (dys)function (16). Azevedo et al. differentiated two newly generated MSA iPSC lines, one MSA-C and one MSA-P, into oligodendrocyte progenitor cells (OPCs) to perform for the first time a whole transcriptomic analysis, identifying an antigen-presenting phenotype in OPCs that impaired their maturation (17). This is a valuable but limited collection of MSA patients-derived iPSC. In the present study, we report the derivation and characterization of six new iPSC lines, five of which come from neuropathologically confirmed MSA patients. With these new lines we aim to contribute to the global effort of generating a faithful MSA human cell model by increasing the number of patients and overcoming their individual genetic background.

## 2 Materials and methods

### 2.1 Patient selection and fibroblasts derivation

Skin biopsies from six age- and gender-matched subjects were obtained through the Catalan MSA Registry (CMSAR) in Hospital Clínic de Barcelona. Five of the subjects had pathologically confirmed MSA, three with the parkinsonian variant and two with the cerebellar variant. The sixth case was diagnosed as clinically established MSA-C. All cases were graded for histopathological severity according to the Jellinger and Seppi classification, which assesses olivopontocerebellar atrophy (OPCA) and striatonigral degeneration (SND) on a scale from I (mild) to III (severe). Demographic and clinical details are provided in **Table 1**. The derivation of human adult dermal fibroblasts (HDF-ad) from a skin biopsy was initiated by washing the collected piece of tissue with Dulbecco’s phosphate buffered saline (DPBS; ThermoFisher Scientific) and cutting it into small pieces. These pieces were transferred into gelatin-coated culture flasks with fibroblast medium, containing 88% Dulbecco’s Modified Eagle’s Medium (DMEM) supplemented with 10% fetal bovine serum (FBS), 1% penicillin/streptomycin (P/S), 0.1 mM non-essential aminoacids solution (NEAA) and 55 µM β-mercaptoethanol (BME; all from ThermoFisher Scientific). During the following 5–8 days, medium changes were performed every other day to maintain optimal conditions and cell growth was monitored. When reaching confluence, fibroblasts were trypsinized and resuspended in cryopreservation medium (50% culture media, 40% FBS and 10% DMSO), this was referred to as passage 0 (P = 0).

**Table 1:** Clinical data of the six selected MSA patients.

iPSC code	Case code	Sex	Variant	1st symptom	Age onset	Age HDF	Time to wheelchair	OH	UI	Stridor	Survival	Pathology
MSA01	MSA006	F	MSA-C	Ataxia	49	62	10	No	No	Yes	15	OPCA III SND II-III + NFT BRAAK I THAL 2 + SVD MILD
MSA02	MSA021	M	MSA-P	Micrographia	66	74	5	Yes	Yes	No	8	OCPA I-II SND III + CTE HIGH GRADE + AD A2B2C2 + CVD/SVD
MSA03	MSA035	F	MSA-P	Slowness	63	61	4	Yes	Yes	Yes	8	OPCA II SND II-III + AD A1B2C0 + SVD MILD
MSA04	MSA038	M	MSA-P	Slowness	71	77	4	Yes	Yes	Yes	7	SND II/III OPCA I-II/III
MSA05	MSA043	F	MSA-C	Dysphagia	56	61	4	Yes	Yes	No	8	NO
MSA06	MSA045	M	MSA-C	Ataxia	63	74	10	Yes	Yes	Yes	11	OPCA II SND II + AD A2B1C0 + MINIMAL SVD

It includes (1) the sex; (2) the MSA variant based on the predominant motor phenotype: MSA-parkinsonism, MSA-P or MSA-cerebellar, MSA-C; (3) the first motor symptom detected; (4) the patient age when experiencing the first symptom(s); (5) the patient age when donating the skin biopsy; (6) the time after the diagnosis when the patient needed a wheelchair, measured in years; (7) the presence of clinical features of autonomic dysfunction like orthostatic hypotension (OH), urinary incontinence (UI) and/or stridor; (8) the survival time after diagnosis, measured in years; and (9) the morphologic postmortem analysis staging that describes the primary and the comorbid neuropathologies: MSA pathology following Jellinger and Seppi classification for olivopontocerebellar atrophy (OPCA) and striatonigral degeneration (SND), ranging from I (mild), II (moderate) and III (severe); Alzheimer’s pathology following the National Institute on Aging-Alzheimer’s Association (NIA-AA) classification; small vessel disease (SVD); and chronic traumatic encephalopathy (CTE).

### 2.2 hiPSCs generation from fibroblasts

Patients-derived skin fibroblasts were thawed and expanded in fibroblast medium. Fibroblasts at low passage (P = 1 or 2) were reprogrammed with the Cytotune-iPS 2.0 Sendai Reprogramming kit (ThermoFishe Scientific), following the manufacturer’s protocol. The kit provides the non-integrative and zero-footprint Sendai virus (SeV) expressing Klf4, Oct3/4, Sox2 and c-Myc transcription factors. Briefly, one well of fibroblasts was used to count cells and perform viral calculations, taking into account the target MOI and the viral titer. Then, the SeV mix was prepared in fibroblast medium and added to a new well of cultured fibroblasts. The following day, the viral medium was replaced by fresh fibroblast medium. On day 7, reprogrammed fibroblasts were plated on irradiated mouse embryonic fibroblasts (iMEFs) using fibroblast medium. On day 8, fibroblast medium was replaced by iPSC medium, containing 78% Dulbecco’s modified Eagle medium/nutrient mixture F-12 (DMEM/F12) supplemented with 20% KnockOut™ serum replacement (KSR), 1% P/S, 0.1 mM NEAA, 55 µM BME and 4 ng/ml basic fibroblast growth factor (bFGF) (all from ThermoFisher Scientific). Three to four weeks after transduction, iPSC colonies had the appropriate size for transfer. They were selected based on their morphology, as they needed to fulfill the following requirements: compact colonies with well-defined and refractive edges, and comprised of cells with high nucleus:cytoplasm ratio. Around 8 passages were performed for each iPSC clone to completely remove the SeV vectors. At first, the iPSC colonies were manually split by cutting them into small pieces in a grid-like pattern and transferring them on iMEFs plates with iPSC medium. Then, iPSC colonies were progressively adapted to 0.5 mM ethylenediaminetetraacetic acid (EDTA, ThermoFisher Scientific) treatment for dissociating them into small clumps and to feeder-free conditions using plates coated with Matrigel (Corning) at a concentration of 8.3 µg/cm^2^ and with mTeSR™ medium (STEMCELL Technologies).

### 2.3 Pluripotency markers validation

#### 2.3.1 Immunofluorescence and confocal microscopy analysis

Cells were fixed with 4% paraformaldehyde (PFA; ThermoFisher Scientific) in DPBS for 10 min at room temperature (RT) and washed twice with DPBS. Then, cells were permeabilized with 0.3% Triton X-100 (Sigma-Aldrich) in DPBS for 10 min and blocked with 3% bovine serum albumine (BSA; Sigma-Aldrich) in DPBS for 1 hour, both steps were performed at RT. Cells were stained with the primary antibody, which was diluted with blocking buffer (0.15% Triton X-100 and 1.5% BSA in DPBS), and incubated overnight at 4 °C. After washing with DPBS, cells were stained with the corresponding secondary antibody in blocking buffer, for 2 hours at RT in the dark. Samples were washed with DPBS and nuclei were stained with 1 µg/ml of 4′,6-diamidino-2-phenylindole (DAPI; ThermoFisher Scientific) for 5 min at RT. Images were captured with an LSM 980 confocal microscope running Zen Black software (Carl-Zeiss) and analyzed with FIJI (ImageJ) software version 2.14.0/1.54f.

The primary antibodies used to assess cell pluripotency were the following: Nanog homeobox (NANOG; Abcam, 1:500) and lin-28 homolog A (LIN28A; Abcam, 1:500). The secondary antibodies were the following, respectively: Alexa Fluor 488 goat anti-mouse (Abcam, 1:500) and Alexa Fluor 647 donkey anti-rabbit (Abcam, 1:500).

#### 2.3.2 Immunofluorescence and flow cytometry analysis

iPSC colonies were dissociated as single cells by the following sequential treatment: first, 1 mg/ml of dispase II (Sigma-Aldrich) in DPBS at 37 °C for about 10 min, when the edges of the colonies started to fold away from the plate surface; and then, TrypLE™ express 1x (ThermoFisher Scientific) at 37 °C for about 3 min, when it was possible to distinguish the individual cells within the colonies. The cell suspension was pipetted up and down, and cells were counted to get 1·10^5^ cells per fluorescence-activated cell sorting (FACS) sample. Cells were centrifuged and resuspended in cold FACS buffer, containing 0.5% BSA, 2 mM EDTA and CEPT in DPBS. CEPT is the combination of 50 nM Chroman 1 (MedChem Express), 5 µM Emricasan (SelleckChem), 1x Polyamine supplement (Sigma-Aldrich) and Trans-integrated stress response inhibitor (trans-ISRIB; R&D systems), and it enhances the cell survival of iPSC by simultaneously blocking several stress mechanisms (18). The cell suspension was filtered using a 70 µM strainer (Corning), and incubated with the SSEA-4-PE/Cyanine7 antibody (Biolegend, 1:500) for 1 hour on ice in the dark. Cells were washed and resuspended with cold FACS buffer containing 1 µg/ml of DAPI. Cells were analyzed with the LSRII Fortessa Cell Analyser (BD Biosciences) and FlowJo software version 10.5.2.

### 2.4 Trilineage differentiation

#### 2.4.1 Monolayer-based trilineage differentiation (early stages validation)

The trilineage developmental potential of iPSCs was assessed using the STEMdiff™ Trilineage Differentiation kit (STEMCELL Technologies), following the manufacturer’s protocol. Specifically, iPSC colonies were passaged as single cells using the Gentle Cell Dissociation Reagent (STEMCELL Technologies) and were plated at a density of 50,000 cells/cm^2^ for mesoderm and 200,000 cells/cm^2^ for ectoderm and endoderm. All conditions were cultured with mTeSR containing CEPT. The following day, the medium was replaced by the appropiate STEMdiff™ Trilineage medium. Medium was changed every day, until day 5 for endoderm and mesoderm and day 7 for ectoderm. Samples were fixed and stained as previously described in the «Pluripotency markers validation. Immunofluorescence and confocal microscopy analysis» section. The primary antibodies used to assess ectoderm-lineage formation were paired box 6 (PAX6; Cell Signaling, 1:100) and nestin (NES; R&D systems, 1:30). For endoderm, SRY-box transcription factor 17 (SOX17; Abcam, 1:50) and forkhead box A2 (FOXA2; Santa Cruz Biotechnology, 1:50). For mesoderm, T-box transcription factor T (TBXT; R&D systems, 1:100) and neural cell adhesion molecule 1 (NCAM1; Abcam, 1:50). The secondary antibodies were Alexa Fluor 488 goat anti-mouse (Abcam, 1:500), Alexa Fluor 555 donkey anti-goat (Abcam, 1:500) and Alexa Fluor 647 donkey anti-rabbit (Abcam, 1:500). Images were captured with an LSM 980 confocal microscope running Zen Black software (Carl-Zeiss) and analyzed with FIJI (ImageJ) software 2.14.0/1.54f.

#### 2.4.2 Embryoid bodies-based trilineage differentiation (late stages validation)

The trilineage developmental potential of iPSC was also assessed by generating embryoid bodies (EB) and culturing them with defined trilineage differentiation media for 3–4 weeks. First, iPSC colonies were dissociated into a cell-cluster suspension (about 3–10 cells), by a sequential treatment with 1 mg/ml Collagenase Type B (Sigma-Aldrich) for 20 min, and 1x TrypLE™ express enzyme for 2 min, both at 37 °C. The resulting cell clusters were cultured (1:2) in ultra-low attachment well plates (Corning) with mTeSR™ medium supplemented with CEPT. After 2–3 days in suspension, EBs were transferred independently using wide-bore pipette tips (ThermoFisher Scientific) into microchambered coverslips (Ibidi), which were previously coated with gelatin for endoderm and mesoderm differentiation, and with matrigel for ectoderm differentiation, and contained the corresponding differentiation medium. The endoderm differentiation medium was: 90% DMEM, 10% FBS, 1% NEAA, 1% GlutaMax, 1% P/S, 0.1 mM BME and 100 ng/ml Activin A (ThermoFisher Scientific) (19). The mesoderm differentiation medium was: 90% DMEM, 10% FBS, 1% NEAA, 1% GlutaMax, 1% P/S, 0.1 mM BME and 100 µM ascorbic acid (AA; Sigma-Aldrich) (19, 20). The ectoderm differentiation medium was: 48% DMEM/F12, 48% Neurobasal, 1% GlutaMax, 1% P/S, 1% N2 supplement and 0.5% B27 supplement (all from ThermoFisher Scientific). EBs were cultured during 3 to 4 weeks and the medium was changed every other day. Samples were fixed and stained as previously described in the «Pluripotency markers validation. Immunofluorescence and confocal microscopy analysis» section. The primary antibody used to assess endoderm was alpha fetoprotein (AFP; Santa Cruz Biotechnology, 1:50); for mesoderm, actin alpha 2 smooth muscle (ACTA2; Abcam, 1:100); and for ectoderm, tubulin beta 3 class III (TUBB3; Sigma-Aldrich, 1:500). The secondary antibody was Alexa Fluor 488 goat anti-mouse (Abcam, 1:500). Images were captured with an LSM 980 confocal microscope running Zen Black software (Carl-Zeiss) and analyzed with FIJI (ImageJ) software version 2.14.0/1.54f.

### 2.5 G-banding karyotype

Chromosome harvest was performed when iPSC colonies reached about 60% of confluency. The mitotic spindle inhibitor Colcemid™ (ThermoFisher Scientific) was added in the culture media at a concentration of 0.1 µg/ml during 2 hours. Then, the supernatant was collected. iPSCs were dissociated into single cells using TrypLE Express, and the cell suspension was combined with the supernatant. The mixture was centrifuged and the pellet was incubated with an hypotonic solution of 0.075 M potassium chloride (KCl; Sigma-Aldrich) at 37 °C for 20 min. Cold Carnoy’s fixative solution I (3 parts of methanol and 1 part of glacial acetic acid, both Sigma-Aldrich) was added at a ratio of 5:1 (hypotonic:fixative), and was incubated for 15 min at RT. The chromosome suspension was centrifuged and underwent some additional fixative incubations: (*i*) cold Carnoy’s fixative solution I for 10 min at RT, two repetitions; and (*ii*) cold Carnoy’s fixative solution II (2 parts of methanol and 1 part of glacial acetic acid) overnight at 4 °C. A small amount of the chromosome suspension was dropped onto slides. Cytogenetics was conducted according to the standard methods used in Laboratori de Citogenètica at Hospital del Mar (Barcelona). Slides were stained using Wright solution (G-banding). A minimum of 20 metaphase were automated screened with Cytoinsight GSL imaging (Leica Biosystems), evaluated with Cytovysion software and informed according to the International System for Human Cytogenetic Nomenclature (ISCN 2024).

### 2.6 Digital PCR for aberrant genomic abnormalities

The Copy Number Variation (CNV) assay was designed based on Assou et al., 2020 publication (21). iPSC at passage 8 were harvest and stored as a dry pellet at -20 °C. Genomic DNA was extracted using the column-based PureLink™ Genomic DNA Kit (ThermoFisher Scientific). DNA concentration and purity were determined with the NanoDrop spectrophotometer (ThermoFisher Scientific), by measuring the absorbance at 260 nm and calculating the 260/280 and 260/230 nm absorbance ratios, respectively. The digital PCR mix was prepared by combining 100 ng of genomic DNA, 10 units of the restriction enzyme HindIII (New England Biolabs), the Absolute Q™ DNA digital PCR master mix 1x (ThermoFisher Scientific), the target TaqMan™ copy number assay primer labeled with FAM fluorophore 1x (ThermoFisher Scientific, **Table 2**), and the reference TaqMan™ copy number assay primer labeled with VIC fluorophore 1x (ThermoFisher Scientific, **Table 2**). The genomic DNA was digested during the reaction set-up period. This digestion was required for the separation of duplications within genomic proximity, enabling their correct identification. The restriction enzyme was inactivated during the first PCR denaturation step. The PCR reactions were loaded in the microfluidic array plate (MAP, ThermoFisher Scientific), and the Absolute Q™ isolation buffer (ThermoFisher) was added on top of each well to prevent contamination and evaporation. The plate was loaded into the QuantStudio Absolute Q™ Digital PCR System (ThermoFisher Scientific) and the amplification conditions were as follows: 96 °C for 10 min; 40 cycles of 96 °C for 5 sec and 60 °C for 15 sec; and hold at 4 °C. Copy number was assessed using the QuantStudio software. Different quality control parameters were checked to make sure that the digital PCR fulfilled within the performance expectations: (*i*) the total number of analyzed micro-chambers per sample was > 20,000; (*ii*) ROX, the reference dye, was uniformly distributed and showed a coefficient of variation (CV) < 11%; and (*iii*) it was possible to define a clear threshold to separate the positive and the negative population and, thus, the “rain” effect was minimum.

**Table 2:** Taqman probes for detecting the six most common abnormal regions in pluripotent stem cells.

Gene	ThermoFisher assay ID	Fluorophore	Loci	Chromosome location	Product size	Compatible restriction enzyme
ID1	Hs01892845_cn	FAM	20q11	hg38|31605682-31605761	79 bp	MseI, HindIII, CviQI
NCAPD2	Hs00509738_cn	FAM	12p13	hg38|6528870-6528956	86 bp	HaeIII, AluI, HindIII, CviQI
STS	Hs00091141_cn	FAM	Xp22	hg38|7350117-7350227	110 bp	MseI, HindIII, CviQI
RPS6KB1	Hs01317454_cn	FAM	17q23	hg38|59910637-59910747	110 bp	HaeIII, AluI, HindIII, CviQI
SOAT1	Hs06606603_cn	FAM	1q25	hg38|179355957-179356068	111 bp	HaeIII, HindIII, CviQI
PITX1	Hs02789072_cn	FAM	5q31	hg38|135029257-135029339	82 bp	MseI, HindIII
RPP30	APU7CK3	VIC	10q23	hg19|chr10:92660373-92660495	67 bp	HaeIII, MseI, AluI, HindIII, CviQI

The target genes (*ID1*, *NCAPD2*, *STS, RPS6KB1*, *SOAT1* and *PITX1*) and the reference gene (*RPP30*) used in the digital PCR copy number assay to identify aberrant duplications in the genomic DNA of the generated iPSC clones. bp (base pair).

### 2.7 Short tandem repeat analysis

Short Tandem Repeat Analysis (STR) for cell line authentication was conducted by Plataforma de Genomica Translacional at Institut d’Investigacio Germans Trias i Pujol (IGTP, Badalona). AmpFLSTR™ Identifiler™ Plus PCR Amplification Kit (ThermoFisher Scientific) was used to determine the number of allele repeat units in microsatellite regions of both the generated iPSC and their parental fibroblast lines. In a first step, a multiplexed PCR of 16 human loci was performed to amplify the amelogenin gender-determining marker and the following tetranucleotide STR markers: D21S11, CSF1PO, vWA, D8S1179, TH01, D18S51, D5S818, D16S539, D3S1358, D2S1338, TPOX, FGA, D7S820, D13S317, D19S433. The amplification conditions were: 95 °C for 11 min; 28 cycles of 94 °C for 20 sec and 59 °C for 3 min; 60 °C for 10 min and hold at 4 °C. Prior to electrophoresis, samples were prepared by adding the PCR product or the allelic ladder to a Hi-Di formamide - GeneScan™ 500 LIZ™ dye size standard (ThermoFisher Scientific) solution. This mixture was denatured at 95 °C for 3 min and snap-cooled. Samples were injected at 3 kV for 10 sec and electrophoresed at 15 kV for 1500 sec in Performance Optimized Polymer-7 (POP-7 polymer; ThermoFisher Scientific) with a run temperature of 60 °C. The PCR products and the allelic ladder were separated and detected by high-resolution capillary electrophoresis with the ABI PRISM 3130xl genetic analyzer (ThermoFisher Scientific). The resulting electropherograms were analyzed using the GeneMapper ID software v3.2 (ThermoFisher Scientific).

### 2.8 Mycoplasma testing

An aliquot of the supernatant, after 48 h culture, was taken. It was incubated at 95 °C for 5 min and centrifuged at 10,000 xg for 30 sec. The PCR reaction was prepared by combining 2 µl of the supernatant, the DreamTaq Green PCR Master Mix 1x (ThermoFisher Scientific), the mycoplasma primers, and the internal control (Qiagen). The primers target the highly conserved 16S rRNA sequences to detect *M. pneumoniae*, *M. hominis*, *M. fermentans*, *U. urealyticum*, *M. pulmonis*, *M. arthritidis*, *M. neurolyticum*, *M. muris* and *M. collis*, which are common human and rodent mycoplasmal species (22). Primer sequences are provided in **Table 3**. A positive mycoplasma control (InvivoGen) was run alongside the iPSC samples. The PCR conditions were as follows: 95 °C for 3 min; 35 cycles of 95 °C for 30 sec and 55 °C for 30 sec and 72 °C for 1 min; 72 °C for 5 min and hold at 4 °C. The PCR products were separated by agarose gel electrophoresis.

**Table 3:** Primer sets used in hiPSC quality control assays.

Target	Primer forward	Primer reverse	Product size
Sendai transgenes screening			
SeV	GGATCACTAGGTGATATCGAGC	ACCAGACAAGAGTTTAAGAGATATGTATC	181 bp
KOS	ATGCACCGCTACGACGTGAGCGC	ACC TTG ACA ATC CTG ATG TGG	528 bp
Klf4	TTCCTGCATGCCAGAGGAGCCC	AATGTATCGAAGGTGCTCAA	410 bp
c-Myc	TAACTGACTAGCAGGCTTGTCG	TCCACATACAGTCCTGGATGATGATG	532 bp
Mycoplasma testing			
Myco	GGGAGCAAACAGGATTAGATACCCT	TGCACCATCTGTCACTCTGTTAACCTC	265–311 bp

(*i*) Four primer sets used to detect the expression of the SeV-derived transgenes in the iPSC lines. The source is the CytoTune-iPS 2.0 Sendai Reprogramming Kit Manual from Invitrogen-ThemoFisher Scientific. (*ii*) A primer set used to detect the highly conserved 16S rRNA sequences, which belong to *M. pneumoniae*, *M. hominis*, *M. fermentans*, *U. urealyticum*, *M. pulmonis*, *M. arthritidis*, *M. neurolyticum*, *M. muris* and *M. Collis.* bp (base pair).

### 2.9 Quantitative RT-PCR for sendai vectors screening

The clones were analyzed by real time RT-PCR to check that they were viral-free iPSCs. 7 days post-transduction fibroblast cells (positive control) and iPSCs at passage 8 (interrogated material for Sendai removal) were harvest (*n* = 3) and stored as a dry pellet at -20 °C. Total RNA was extracted using the MagMAX−96 Total RNA Isolation Kit (ThermoFisher Scientific). RNA concentration and purity were determined with the NanoDrop spectrophotometer (ThermoFisher Scientific), by measuring the absorbance at 260 nm and calculating the 260/280 and 260/230 nm absorbance ratios, respectively. 1 µg of RNA was treated with the ezDNase enzyme (ThermoFisher Scientific) for genomic DNA removal and reverse-transcribed with the Superscript IV VILO reverse transcriptase (ThermoFisher Scientific). Real time quantitative PCR was performed on a ViiA 7 Real-Time PCR System with OptiFlex Optics System (ThermoFisher Scientific) using PowerUp SYBR Green PCR kit (ThermoFisher Scientific). The PCR reactions were conducted using the following parameters: 95 °C for 2 min; and 40 cycles of 95 °C for 30 sec, 60°C for 30 sec and 72°C for 30 sec. Primer sequences are provided in **Table 3**. Gene expression levels relative to the glyceraldehyde 3-phosphate dehydrogenase (*GAPDH*) housekeeping gene were calculated by the 2^−ΔΔCt^ method, the Livak method (23). For each patient-derived cell line, the 7 days post-transduction fibroblast population was used as the reference sample. The expression value of the interrogated sample (iPSC population at passage 8) was normalized to this reference sample, giving an expression value relative to 1. The log10 of the relative normalized expression was calculated, and the data was displayed using the Box and Whisker plots.

### 2.10 Directed differentiation to oligodendrocytes

iPSCs were differentiated to oligodendrocytes as previously described in Douvaras et al., 2015 protocol (24), with some modifications. iPSCs were dissociated with Accutase (ThemoFisher Scientific) as single cells and plated on Matrigel-coated 6-well plates with mTeSR and CEPT at a density between 100,000 - 400,000 cells/well depending on the cell line. This cell density was adjusted for each iPSC line to ensure that in 1–2 days the colonies could reach a diameter of 100 - 250 µm. mTeSR medium was replaced by Neural Induction Media, NIM [DMEM/F12 containing 10 μM SB431542 (Stemgent), 250 nM LDN193189 (Stemgent) and 100 nM all-*trans* retinoic acid (RA, Sigma-Aldrich)]. After 8 days of culture, NIM medium was switched to N2 medium [DMEM/F12 containing 1x N2 supplement, 100 nM RA and 1 μM smoothened agonist (SAG, Sigma-Aldrich)]. NIM and N2 medium promote the specification of the progenitor motor neuron (pMN) domain. On day 12, cells were overconfluent and piled up generating 3D structures. At this point, cells were mechanically dissociated with a cell scraper (VWR) to obtain spheres in suspension, which were cultured (1:2) in ultra-low attachment 6-well plates with N2B27 medium [DMEM/F12 containing 1x B27 without vitamin A supplement, 1x N2 supplement, 100 nM RA and 1 μM SAG]. Only oligodendrocyte lineage transcription factor 2-positive (OLIG2+) cells are able to form aggregates and, thus, OLIG2- cells are eliminated gradually during medium changes. On day 20, N2B27 media was switched to PDGF media, based on DMEM/F12 supplemented with 1x B27 without vitamin A supplement, 1x N2 supplement, 10 ng/ml platelet-derived growth factor AA (PDGFAA; R&D systems), 10 ng/ml insulin-like growth factor 1 (IGF-I; R&D systems), 5 ng/ml hepatocyte growth factor (HGF; R&D systems), 10 ng/ml neurotrophin 3 (NT3; Sigma-Aldrich), 60 ng/ml 3,3,5-Triiodo-L-thyronine (T3; Sigma-Aldrich), 100 ng/ml biotin (Sigma-Aldrich), 1 µM N6,2′-O-Dibutyryladenosine 3′,5′-cyclic monophosphate sodium salt (cAMP analog; Sigma-Aldrich), and 25 µg/ml insulin solution (Sigma-Adrich). These recombinant proteins and chemical compounds drive oligodendrocyte survival and differentiation. On day 30, spheres with round shape, brown color and a diameter of 300 - 800 µm were manually picked using a microscope under sterile conditions and a p200 pipette with a wide-bore tip. The selected spheres were plated on poly-L-ornithine/laminin-coated 6-well plates with PDGF medium, at a concentration of 2 spheres per cm^2^. Cells migrated out of the spheres, first neurons and astrocytes, and around day 50 oligodendrocytes. From day 75 to 90, cells were maintained with glial medium [DMEM/F12 containing 1x B27 without vitamin A supplement, 1x N2 supplement, 10 mM HEPES (Sigma-Aldrich), 60 ng/ml T3, 100 ng/ml biotin, 1 µM cAMP, 25 µg/ml insulin solution, and 20 µg/ml AA] to induce the terminal differentiation of OPCs to more mature OLCs.

At day 75, the immunofluorescence and cell sorting of OPCs with the oligodendrocyte-specific O4 antibody was done with live cells. For immunoflurescence, the O4 primary antibody (R&D systems, 1:50) and 5% goat serum were added into the culture medium. Cells were incubated at 37 °C for 45 min. A wash step with DMEM/F12 was performed. Cells were fixed with 4% PFA for 10 min and the protocol was continued as previously described. A goat anti-mouse secondary antibody conjugated with Alexa Fluor 488 (ThermoFisher, 1:500) was used. For FACS, cells were dissociated with Accutase as single cells. The spheres remained intact after the Accutase treatment, but we were only interested in obtaining the cells that had migrated out of these spheres. Cells were centrifuged and incubated with DMEM/F12 medium containing the O4 primary antibody and 5% goat serum for 45 min on ice. A wash step with DMEM/F12 was performed. Cells were centrifuged and resuspended with DMEM/F12 medium containing the goat anti-mouse secondary antibody conjugated with Alexa Fluor 488 for 25 min on ice. Cells were washed, resuspended with cold PDGF medium and filtered with a 70 µM strainer. During this protocol, the cell suspension was pipetted up and down very gently a maximum of 2–3 times/step to avoid high cell death. Cells were sorted using a BD FACSAria II or a BD Influx (BD Biosciences).

At day 90, samples were fixed and stained as previously described in the «Pluripotency markers validation. Immunofluorescence and confocal microscopy analysis» section. The primary antibodies were the following: for neurons, microtubule-associated protein 2 (MAP2; Abcam); for astrocytes, glial fibrillary acidic protein (GFAP; Dako); for OLCs, myelin basic protein (MBP; Sigma-Aldrich). The corresponding secondary antibodies were: Alexa Fluor 647 goat anti-chicken (ThermoFisher Scientific, 1:500), Alexa Fluor 488 donkey anti-rabbit (ThermoFisher Scientific, 1:1000) and Alexa Fluor 568 goat anti-rat (ThermoFisher Scientific, 1:1000) respectively. Images were captured with an LSM 980 confocal microscope running Zen Black software (Carl-Zeiss) and analyzed with FIJI (ImageJ) software version 2.14.0/1.54f. All reagents and antibodies used in this article are listed in **Table 4**.

**Table 4:** Key resource table.

Reagent	Source	Identifier
Dulbecco’s modified Eagle’s medium (DMEM), high glucose	Gibco-ThermoFisher Scientific	Cat#31966021
Dulbecco’s modified Eagle medium/nutrient mixture F-12 (DMEM/F12)	Gibco-ThermoFisher Scientific	Cat#10565018
Neurobasal	Gibco-ThermoFisher Scientific	Cat#21103049
Fetal bovine serum (FBS), embryonic stem cell (ESC)-qualified	Gibco-ThermoFisher Scientific	Cat#10270106
KnockOut™ serum replacement (KSR)	Gibco-ThermoFisher Scientific	Cat#10828028
Penicillin/Streptomycin (P/S)	Gibco-ThermoFisher Scientific	Cat#15140122
GlutaMAX	Gibco-ThermoFisher Scientific	Cat#35050061
Non-essential amino acids solution (NEAA)	Gibco-ThermoFisher Scientific	Cat#11140050
b-mercaptoethanol, 55 mM Solution (BME)	Gibco-ThermoFisher Scientific	Cat#21985023
mTeSR™ serum-free medium	STEMCELL Technologies	Cat#85850
N2 supplement (100x)	Gibco-ThermoFisher Scientific	Cat#17502001
B27 supplement (50x)	Gibco-ThermoFisher Scientific	Cat#A1486701
B27 supplement without vitamin A (50x)	Gibco-ThermoFisher Scientific	Cat#12587010
Ethylenediaminetetraacetic acid (EDTA), 0.5 M, pH 8.0	Invitrogen-ThermoFisher Scientific	Cat#15575020
Trypsin-EDTA (0.25%)	Gibco-ThermoFisher Scientific	Cat#25200056
TrypLE™ Express	Gibco-ThermoFisher Scientific	Cat#12604039
Dispasse II	Sigma-Aldrich	Cat#D4693
Collagenase type B	Sigma-Aldrich	Cat#11088807001
Gentle Cell Dissociation Reagent (GCDR)	STEMCELL Technologies	Cat#100-0485
StemPro™ Accutase™ cell dissociation reagent	Gibco-ThermoFisher Scientific	Cat#A1110501
Dulbecco’s phosphate-buffered saline (DPBS), no calcium, no magnesium	Gibco-ThermoFisher Scientific	Cat#14190144
Gelatin, 0.1% (w/v) in water	STEMCELL Technologies	Cat#07903
Matrigel, growth factor-reduced	Corning	Cat#356230
Poly-L-ornithine hydrobromide	Sigma-Aldrich	Cat#P3655
Laminin mouse protein, natural	Gibco-ThermoFisher Scientific	Cat#23017015
Triton X-100	Sigma-Aldrich	Cat#X100
Bovine serum albumine (BSA)	Sigma-Aldrich	Cat#A9418
Chroman 1	MedChem Express	Cat#HY-15392
Emricasan	SelleckChem	Cat#S7775
Polyamine supplement (1000x)	Sigma-Aldrich	Cat#P8483
Trans-integrated stress response inhibitor (trans-ISRIB)	R&D systems	Cat#5284
Ascorbic acid	Sigma-Aldrich	Cat#A4403
Basic fibroblast growth factor (bFGF)	PeproTech-ThermoFisher Scientific	Cat#100-18B
Activin A	PeproTech-ThermoFisher Scientific	Cat#120-14P
SB431542	Reprocell	Cat#04-0010
LDN193189	Reprocell	Cat#04-0074
all-*trans* retinoic acid (RA)	Sigma-Aldrich	Cat#R2625
Smoothened agonist (SAG)	Sigma-Aldrich	Cat#566660
Platelet-derived growth factor AA (PDGFAA)	R&D systems	Cat#221-AA-050
Insulin-like growth factor 1 (IGF-I)	R&D systems	Cat#291-G1-200
Hepatocyte growth factor (HGF)	R&D systems	Cat#294-HG-025/CF
Neurotrophin 3 (NT3)	Sigma-Aldrich	Cat#GF308
3,3,5-Triiodo-L-thyronine (T3)	Sigma-Aldrich	Cat#T2877
Biotin	Sigma-Aldrich	Cat#B4639
N6,2′-O-Dibutyryladenosine 3′,5′-cyclic monophosphate sodium salt (cAMP analog)	Sigma-Aldrich	Cat#D0260
Insulin solution, human	Sigma-Aldrich	Cat#I9278
HEPES	Sigma-Aldrich	Cat#H4034
Restriction enzyme HindIII high fidelity	New England Biolabs	Cat#R3104S
Colcemid™ solution in PBS	Gibco-ThermoFisher Scientific	Cat#15212012
Paraformaldehyde, 4% (w/v) in PBS	ThermoFisher Scientific	Cat#J61899.AP
Dimethyl Sulfoxide (DMSO)	Sigma-Aldrich	Cat#D2650
4′,6-diamidino-2-phenylindole (DAPI)	Invitrogen-ThermoFisher Scientific	Cat#D1306
Cytotune-iPS 2.0 Sendai reprogramming kit	Invitrogen-ThermoFisher Scientific	Cat#A16517
STEMdiff™ trilineage differentiation kit	STEMCELL Technologies	Cat#05230
AmpFLSTR™ Identifiler™ Plus PCR amplification kit	Applied Biosystems-ThermoFisher Scientific	Cat#4427368
PureLink™ genomic DNA Kit	Invitrogen-ThermoFisher Scientific	Cat#K182001
Antibody	Source	Identifier
Rabbit monoclonal anti-paired box 6 (PAX6)	Cell Signaling	Cat#60433; RRID: AB_2797599
Mouse monoclonal anti-nestin (NES)	R&D systems	Cat#MAB1259; RRID: AB_2251304
Goat polyclonal anti-T-box transcription factor T (TBXT)	R&D systems	Cat#AF2085; RRID: AB_2200235
Rabbit monoclonal anti-neural cell adhesion molecule 1 (NCAM1)	Abcam	Cat#AB237708; RRID: AB_3676336
Rabbit monoclonal anti-SRY-box transcription factor 17 (SOX17)	Abcam	Cat#AB224637; RRID: AB_2801385
Mouse monoclonal anti-forkhead box A2 (FOXA2)	Santa Cruz Biotechnology	Cat#sc-271103; RRID: AB_10614496
Mouse monoclonal anti-nanog homeobox (NANOG)	Abcam	Cat#AB173368; RRID: AB_3076592
Rabbit polyclonal anti-lin-28 homolog A (LIN28A)	Abcam	Cat#AB63740; RRID: AB_1310410
Mouse monoclonal anti-tubulin beta 3 class III (TUBB3)	Sigma-Aldrich	Cat#T8660; RRID: AB_477590
Mouse monoclonal anti-alpha fetoprotein (AFP)	Santa Cruz Biotechnology	Cat#sc-130302; RRID: AB_2223934
Mouse monoclonal anti-actin alpha 2, smooth muscle (ACTA2)	Abcam	Cat#AB7817; RRID: AB_262054
Mouse monoclonal anti-oligodendrocyte marker O4 (O4)	R&D systems	Cat#MAB1326; RRID: AB_357617
Mouse monoclonal anti-stage-specific embryonic antigen-4 (SSEA-4)	BioLegend	Cat#330419; RRID: AB_2629630
Rat monoclonal anti-myelin basic protein (MBP)	Sigma-Aldrich	Cat#MAB386; RRID: AB_94975
Chicken polyclonal anti-microtubule associated protein 2 (MAP2)	Abcam	Cat#AB5392; RRID: AB_2138153
Rabbit polyclonal anti-glial fibrilliary acidic protein (GFAP)	Dako Agilent	Cat#Z033401-2; RRID: AB_10013382
Alexa Fluor 488 goat polyclonal anti-mouse	Abcam	Cat#AB150113; RRID: AB_2576208
Alexa Fluor 555 donkey polyclonal anti-goat	Abcam	Cat#AB150130; RRID: AB_2927775
Alexa Fluor 647 donkey polyclonal anti-rabbit	Abcam	Cat#AB150075; RRID: AB_2752244
Alexa Fluor 568 goat polyclonal anti-rat	Invitrogen-ThermoFisher Scientific	Cat#A-11077; RRID: AB_2534121
Alexa Fluor 647 goat polyclonal anti-chicken	Invitrogen-ThermoFisher Scientific	Cat# A-21449; RRID: AB_2535866
Alexa Fluor 488 goat polyclonal anti-mouse	Invitrogen-ThermoFisher Scientific	Cat#A-21042; RRID: AB_2535711
Alexa Fluor 488 donkey polyclonal anti-rabbit	Invitrogen-ThermoFisher Scientific	Cat#A-21206; RRID: AB_2535792

## 3 Results

### 3.1 MSA patients-derived fibroblasts were reprogrammed to iPSC

Six MSA cases were selected from the CMSAR in Hospital Clínic de Barcelona to generate a sex-balanced iPSC collection representing the two disease variants (cerebellar and parkinsonian). For five out of six cases, both the skin biopsy and the brain were available and we could confirm a definitive MSA diagnosis as defined by the postmortem identification of aggregated and misfolded aSyn in OLs forming GCIs. Four cases also presented other comorbid neuropathology. The MSA06 case did not donate the brain but fulfills both Gilman’s 2008 criteria for probable MSA (25) and the Movement Disorder Society (MDS) 2022 criteria for clinically established MSA (26) (**Table 1**).

**Figure 1:**
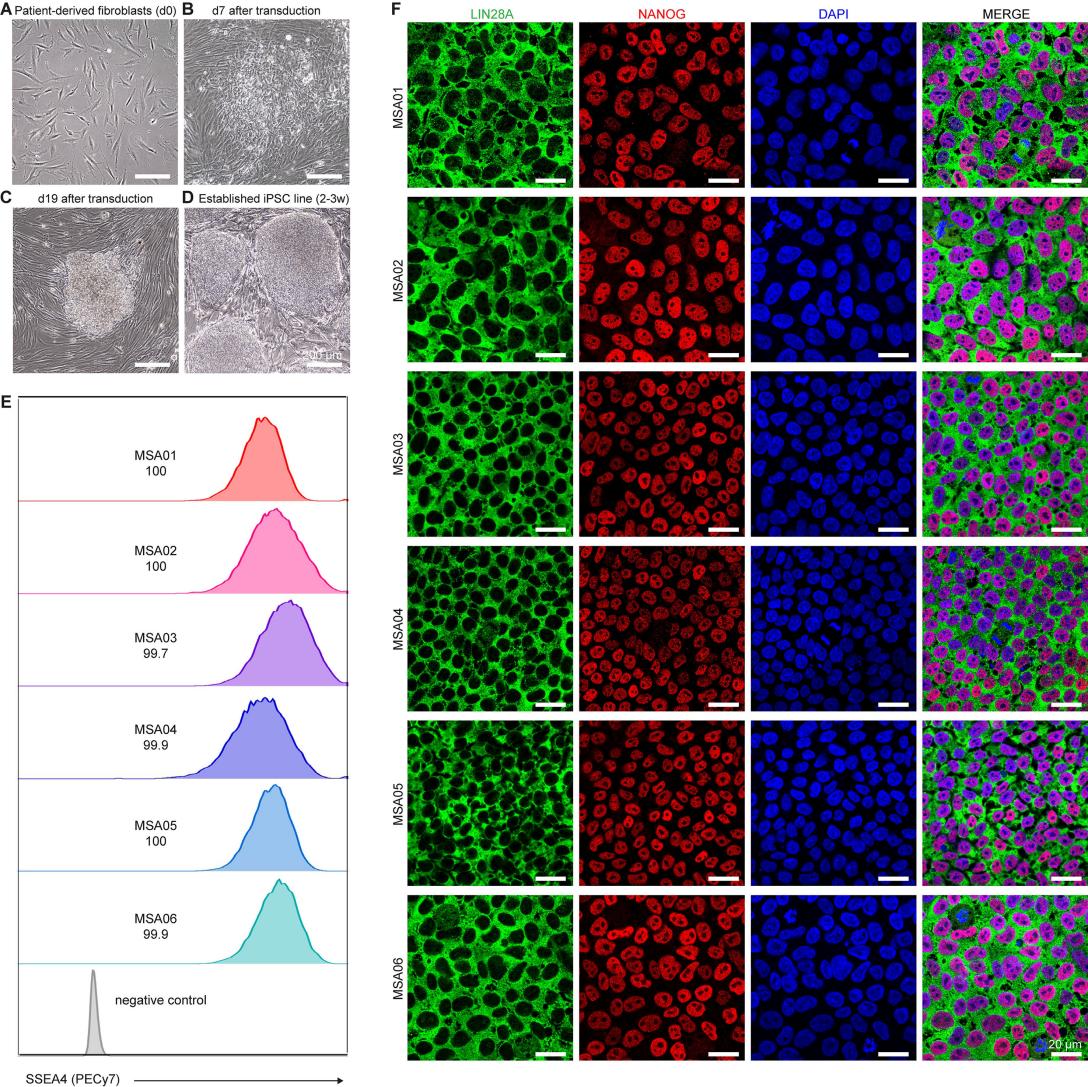
Analysis of the morphology and the pluripotency-associated markers expression of the six MSA patient-derived iPSC lines. **(A-D)** Representative phase contrast microscopy images of the main morphological changes that accompanied the reprogramming to pluripotency: **(A)** the fibroblasts obtained from skin biopsies had an elongated spindle shape; **(B)** 7 days after SeV transduction, some cells started to show a rounder shape and a colony growth pattern; **(C)** 19 days after SeV transduction and 12 days after being cultured on feeder layers, the emergence of iPSC colonies was easily distinguishable, and these cells exhibited an ES-like morphology characterized by a high nucleus to cytoplasm ratio and a cobblestone shape; **(D)** after manually picking single cell colonies and passaging them a minimum of 2–3 times on feeder layers, the iPSC colonies maintained an undifferentiated state, and cells grew in uniform and tightly packed colonies, with well-defined sharp borders. **(E)** Flow cytometry analyses showed the proportion of SSEA4+ cells after establishing the iPSC lines (2–3 weeks of culture). **(F)** The key regulators of pluripotency in mammalian iPSCs, NANOG and LIN28A, were uniformly expressed in all six MSA patient-derived iPSC lines as demonstrated by immunofluorescence. Scale bars **(A-D)** 200 µm; and **(F)** 20 µm.

From the skin biopsies, fibroblast cells were isolated, expanded for two passages, and validated to be mycoplasma-free. HDF-ad appeared spindle-shaped and refractile, with a doubling time of approximately 24 h (**Figure 1A**). HDF-ad were plated to reach 30 - 60% confluency after 48 h, this seeding density was optimized for each patient’s cell line. HDF-ad were reprogrammed using the non-integrative SeV for delivering the transcription factors Oct3/4, Sox2, c-Myc and Klf4 (OSKM, referred to as the Yamanaka factors). After seven days, transduced cells were plated on irradiated mouse embryonic fibroblasts (iMEFs) and cultured with iPSC medium. At this time point, some cells were already experimenting an evident and distinctive morphology change: from the initial elongated morphology (fibroblast-like) to a compact polygonal morphology (epithelial cell-like) (**Figures 1B, C**). Approximately between 3- and 4-week after reprogramming, tightly packed colonies were visible, and were characterized by having well-defined edges and containing cells with a high nucleus-cytoplasm ratio, features also shown in human embryonic stem (ES) cell colonies (**Figure 1D**). Single colonies were picked and clonally expanded, a process during which iPS cells were progressively adapted to mTeSR medium and Matrigel coating.

Our objective was to obtain three independent validated iPSC clones per patient, which met all the pre-defined quality standards (pluripotency expression, genome integrity, and differentiation potential). Some clones could fail in the evaluation process and, thus, a backup repository of nine independent iPSC clones per patient was generated. Then, three clones were selected randomly to evaluate whether they pass the before mentioned quality tests. In this manuscript, we only show the complete dataset of only one validated iPSC clone per patient.

The pluripotency of the newly generated iPSC lines was assessed by studying the expression of a set of signature surface and intracellular pluripotency proteins, SSEA4, NANOG and LIN28A. A combination of quantitative and qualitative data was obtained. The flow cytometry analysis demonstrated that > 99% of the cells were SSEA4+ in all the six iPSC lines (**Figure 1E**), being the acceptance criteria > 70% (27). The image-based analysis of NANOG and LIN28A revealed that these two proteins were expressed simultaneously and homogenously in the *in vitro* culture of all the six iPSC lines (**Figure 1F**).

### 3.2 MSA hiPSC retained genomic integrity and became footprint-free after reprogramming and long-term expansion

**Figure 2:**
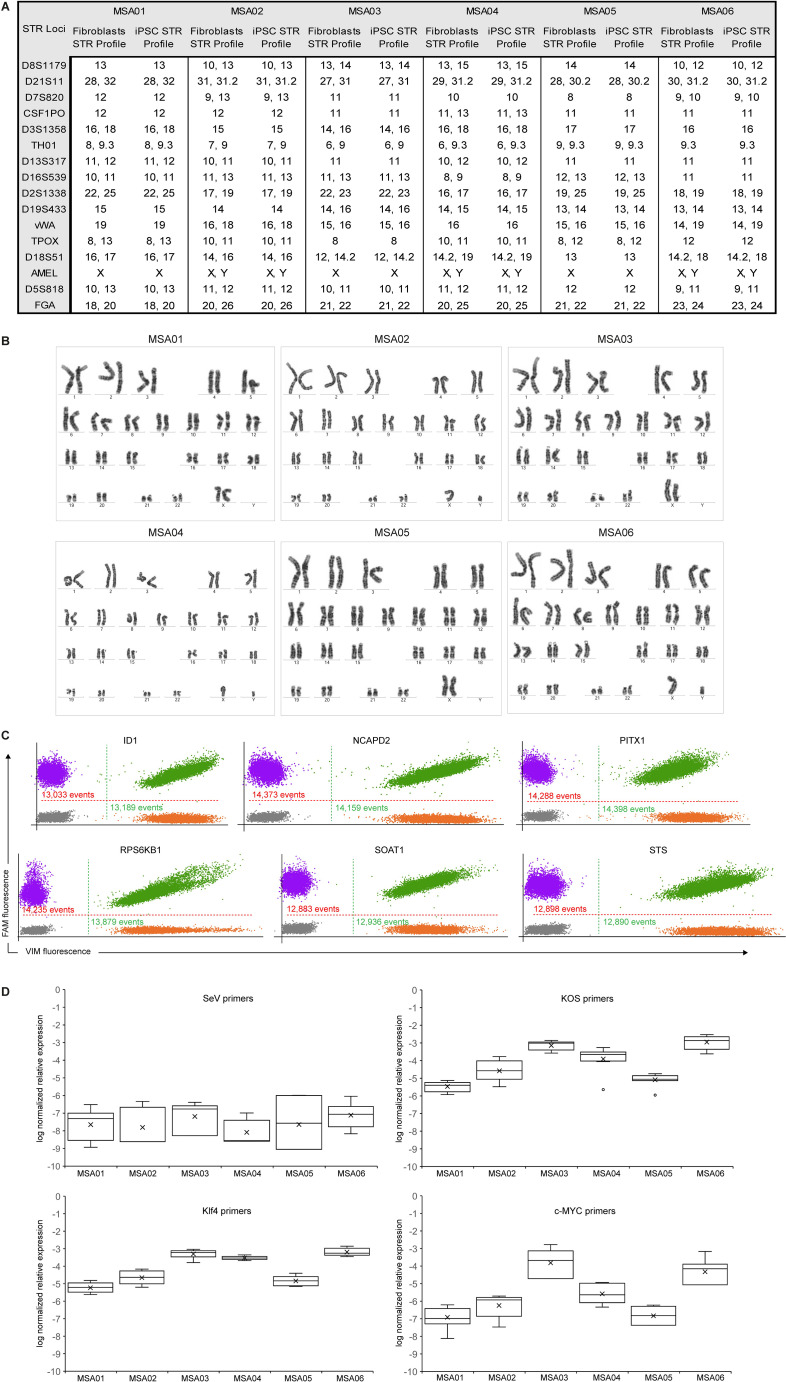
Assessment of the cell line identity and the genomic integrity of the six MSA patient-derived iPSCs. **(A)** Short tandem repeat profiling provided the number of repeating units of 16 polymorphic microsatellite loci, to compare the donor fibroblast cells (reference) and the generated iPSCs and verify their origin. **(B)** G-banding revealed that the six iPSC lines showed a normal diploid karyotype (n = 46) and matched the sex of the donor patient. **(C)** Digital PCR allowed us to screen whether specific genomic regions accumulated duplications that may give a strong selective survival or growth advantage to iPSCs. Example of the scatter digital PCR plots displaying simultaneously (1) FAM signals, shown in purple and green to detect the target gene (*ID1*, *NCAPD2*, *PITX1*, *RPS6KB1*, *SOAT1*, or *STS*), which are located in the 6 most recurrent abnormal regions; and (2) VIM signals, shown in orange and green to detect the reference gene, *RPP30*. The ratio between the number of copies of the target gene and of the reference gene was used to determine the normal 2-copy number or copy number variations. The plots were obtained through QuantStudio 3D Digital PCR system. In all cases, we obtained 2-copy number **(D)** Quantitative Real Time RT-PCR was used for confirming the absence of the Sendai virus in the iPSC lines after 8 passages, through four primer sets (SeV, KOS, Klf4 and c-Myc). Box and Whisker plots depict in log10 scale the gene expression relative to *GAPDH* gene and normalized to the 7 days post-transduction fibroblasts, calculated by the 2(-ΔΔC(T)) method. In all iPSC clones, the expression levels of the SeV RNA were very low, reaching the limit of detection. Data are shown as the mean ± s.d. n = 3.

Reprogrammed MSA hiPSC fully retained their donor-specific pattern of sequence repeats, as confirmed by STR profiling (**Figure 2A**), and thus cell lines identity could be verified with high accuracy. iPSC maintained their normal diploid karyotype after reprogramming and prolonged cell culture (minimum 10 passages), as determined by a 20-metaphase G-banding analysis (**Figure 2B**). G-banding offers whole-genome scans of chromosomal structure and size, but with a limited resolution at the level of 5–10 megabases (28). However, human iPSC and ES tend to accumulate recurrent copy number variations smaller than 5 megabases at specific genome locations (hotspots) (21). They include gains at 20q11.2 (encompassing *ID1*, *BCL2L1, DNMT3* and *HM13* genes) (29–31); 12p13.31 (*NANOG* and *GDF3* genes) (32, 33); and 17q25.3 (*BIRC5* gene) (34). Still under discussion, the amplification of these candidate genes could confer to the abnormal cells an enhanced probability of self-renewal, proliferation and/or survival, rapidly overtaking the culture as an adaptation process (33, 35). For this reason, the genomic DNA of the iPSC cultures at passage 8 was screened by digital PCR, targeting six of the previously identified and described recurrent abnormalities (approximately 60% of cumulated coverage) (21). The iPSC clones had 2 copies of the following genes: *ID1* (20q11 locus), *NCAPD2* (12p13 locus), *STS* (Xp22 locus), *RPS6KB1* (17q23 locus), *SOAT1* (1q25 locus) and *PITX1* (5q31 locus), relative to the reference gene *RPP30* (10q23 locus) (**Figure 2C**). This quality control approach should be routinely repeated when cells are cultured for long-term periods.

The selected reprogramming method was the negative-sense single-stranded RNA SeV since it replicates cytosolically in the host cells and it does not have a DNA phase throughout its life cycle, eliminating the risk of genomic integration of OSKM (36). Real Time RT-PCR using SeV-specific primers was performed to check that the reprogramming transgenes were lost during host cell division. It is a passage-dependent decrease (37), which can be accelerated when iPSC are passaged as a single-colony instead of a bulk-population. Consequently, during the firsts 3 passages, the hiPSC expansion was performed as follows: manually selecting a single colony, cutting it in a grid-like pattern and transferring it into a feeder layer- or matrigel- coated plate. For the rest of the passages, EDTA was used to perform a pooled-clone expansion. MSA hiPSC clones were analyzed at passage 8 with quantitative Real Time RT-PCR, and 7-day transduced cells were used as positive controls. The absence of exogenous viral sequences was confirmed for all cases (**Figure 2D**).

### 3.3 MSA hiPSC demonstrated their differentiation competency

**Figure 3:**
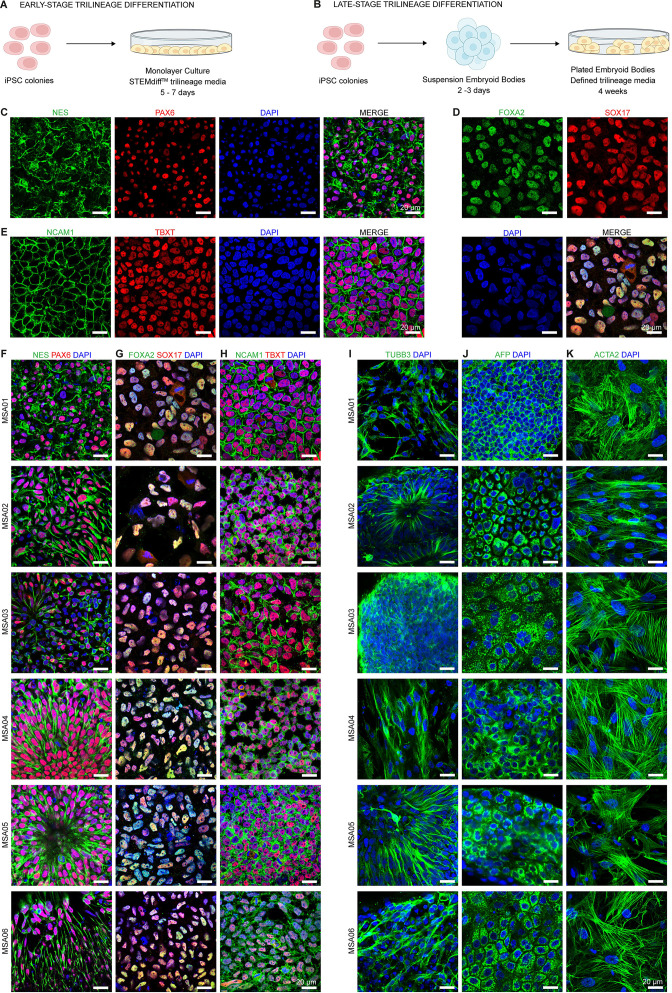
Evaluation of the differentiation potency of the six MSA patient-derived iPSC lines, using two different functional assays that differ in the developmental time: **(A)** early-stage and **(B)** late-stage in the induction of all the three embryonic germ layers. **(A)** Schematic representation of the early-stage method, in which iPSCs were singularized, cultured as a monolayer, and treated with defined differentiation commercial mediums. This protocol is based on the STEMdiff™ Trilineage Differentiation Kit. **(B)** Schematic representation of the late-stage method, in which embryoid bodies were generated, grown during 2–3 days, transferred in adherent conditions, and treated with defined home-made differentiation mediums. The differentiation potential was established **(A)** within 1 week or **(B)** after 4 weeks. **(C-E)** Representative three-channel immunofluorescence imaging and the corresponding merged obtained using the **(A)** method and the MSA01 cell line, **(C)** PAX6 combined with NES as ectoderm markers; **(D)** SOX17 combined with FOXA2 as endoderm markers; and **(E)** Brachyury (TBXT) combined with NCAM1 as mesoderm markers. **(F)** All early ectodermal (PAX6 and NES), **(G)** endodermal (SOX17 and FOXA2), and **(H)** mesodermal (TBXT and NCAM1) lineage markers were homogeneously and highly expressed in the six MSA patient-derived iPSC line. **(I-K)** Equivalently, all late markers were detected. **(I)** The ectoderm derivative showed cells extending projections and ubiquitously expressing the β-tubulin III (TUBB3) protein. Some of these cells were assembled as neural rosettes. **(J)** The induction of the endoderm was confirmed by the expression of α-fetoprotein (AFP), revealing a single layered cup-shaped epithelium. **(K)** For the mesodermal differentiation, cells expressed α-smooth muscle actin (ACTA2), exhibiting the distinctive contractile stress fibrils. Nuclei were counterstained with DAPI in **(C-K)**. Scale bars **(C-K)** 20 µm.

The differentiation potential of the six MSA hiPSC lines was assessed by performing two independent and complementary protocols. Cells were differentiated into the three germ layers (ectoderm, endoderm and mesoderm) by recapitulating early or late stages of the human embryonic development in a dish. First, iPSC were plated as single cells in an adherent monolayer culture and were treated with a commercial, defined and lineage-specific media for 5 or 7 days (STEMdiff™ Trilineage Differentiation Kit from STEMCELL Technologies) (**Figure 3A**). It is a rapid and reproducible method, based on the chemical induction of the early stages of the trilineage differentiation. On the other hand, embryoid bodies (EBs) were generated and plated in gelatin-coated plates for ectoderm and matrigel-coated plates for endoderm and mesoderm. Following this, the EBs were treated with a homemade, defined and lineage-specific media for 4 weeks (**Figure 3B**). EBs are three-dimensional cell clusters that can physically recapitulate embryogenesis. Thus, this protocol allows to better observe the morphogenesis of the ectoderm, endoderm and mesoderm and the prolonged culture time enables the emergence of late stages of the trilineage differentiation. The expression of the three germ-layer proteins was monitored through immunofluorescence in both cases. For the ectoderm, neural tube-like rosettes were clearly recognizable in both early- and late- stage differentiations. The transcription factor PAX6 (38) was expressed uniformly by columnar cells. Also, the cytoskeletal protein NES (39) showed that cells developed processes such as axon-like domains. PAX6 and NES are the predominant markers of neural progenitor cells (**Figure 3C** is exemplary shown for MSA01, **Figure 3F**; **Supplementary Figure 1**). The presence of TUBB3-positive cells (40) also confirmed the neuronal fate commitment (**Figure 3I**). As the differentiation proceeds, this tubulin is expressed by postmitotic immature neurons and, like nestin, is involved in the cytoskeleton remodeling required for neural plasticity. The endoderm formation was demonstrated by the earlier expression of FOXA2 and SOX17 transcription factors that act as epithelial to mesenchymal (EMT) suppressors and as epithelial gatekeepers (41) (**Figure 3D** is exemplary shown for MSA01, **Figure 3G**; **Supplementary Figure 2**). The later expression and localization of the secreted serum protein AFP (42) confirmed the organization of a developing epithelium, characterized by a single layered cup-shaped sheet of cells (**Figure 3J**). For the mesoderm, cells expressed the adhesion molecule NCAM1 and the transcription factor TBXT, both implicated in cell migration and the commitment to the mesodermal lineage, as it is formed via EMT (43) (**Figure 3E** is exemplary shown for MSA01, **Figure 3H**; **Supplementary Figure 3**). After the EB differentiation, a mesenchymal tissue was identified. Specifically, the expression of the actin protein ACTA2 revealed the presence of stress fibers from the derived stromal cells (**Figure 3K**).

### 3.4 MSA hiPSC efficiently generated oligodendrocytes *in vitro*

**Figure 4:**
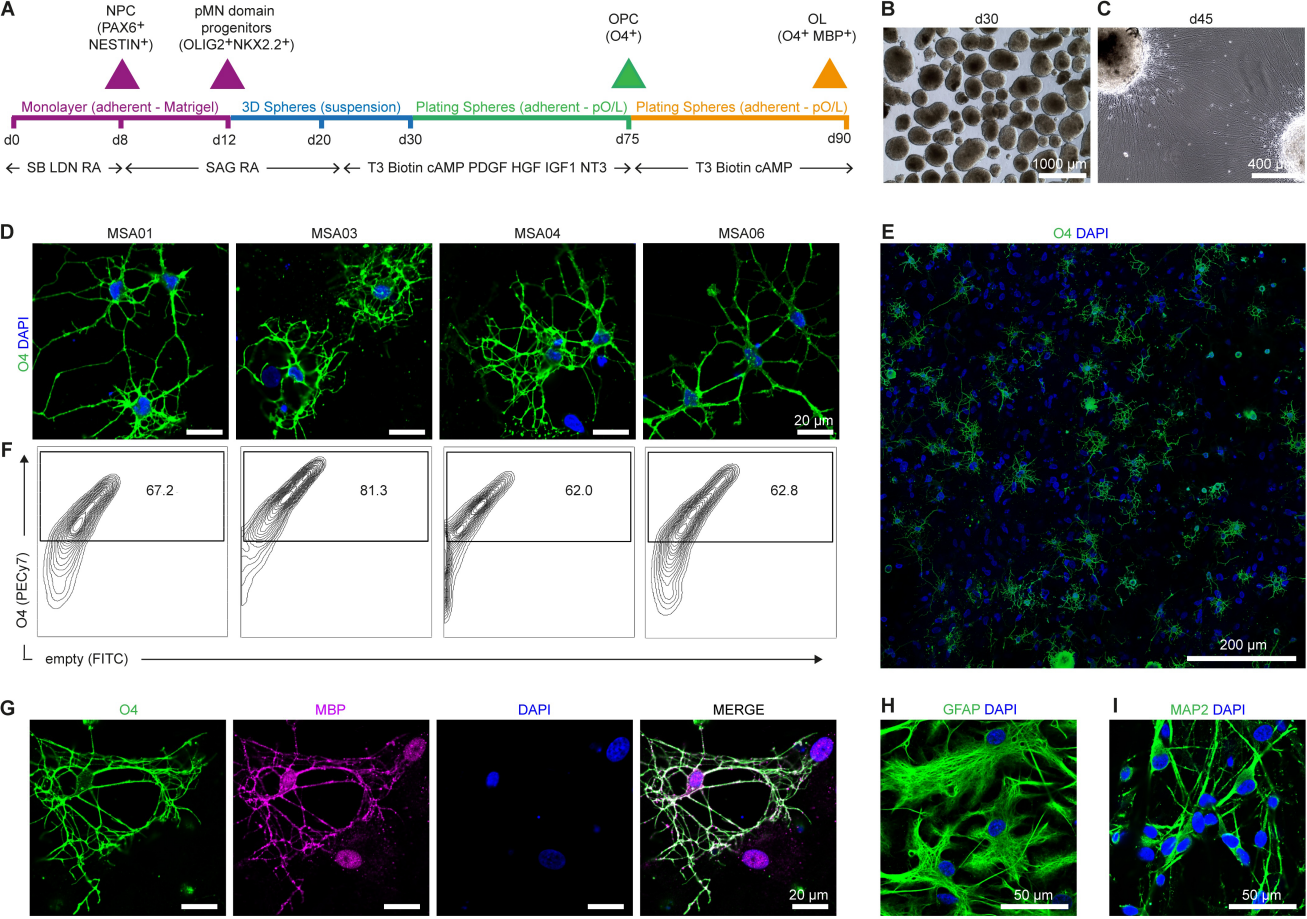
Generation of oligodendrocyte progenitor cells and oligodendrocyte cells from four MSA patient-derived iPSC lines. **(A)** Schematic representation of the protocol followed to differentiate hiPSC into oligodendrocytes. The protocol consists of three stages: (1) induction of the progenitor motor neuron-like domain from days 0–12 in monolayer with the combination of dual SMAD inhibition and RA and SHH activation; (2) specification to oligodendrocyte progenitor cells as neurospheres by treatment with SAG and RA during days 12 - 20, and T3, biotin, cAMP, PDFG, HGF, IGF1 and NT3, days 20 - 75; (3) maturation to oligodendrocyte cells by the withdrawal of PDGF, HGF, IGF1 and NT3 days 75 - 90. **(B, C)** Two distinctive moments of the protocol: **(B)** at day 30, the neurospheres in suspension were round-shaped, with a diameter between 300 - 800 µm and a darker core; **(C)** at day 45, the plated neurospheres into poly-L-ornithine/laminin-coated dishes showed the migration of cells out of the spheres. **(D)** Imaging and quantification of O4+ oligodendrocyte progenitor cells at day 75 for each of the four MSA patient-derived iPSC lines. High magnification images highlighting the branched morphology of oligodendrocytes. **(E)** Representative image at lower magnification demonstrating the generation of an enriched-oligodendrocyte culture. **(F)** Flow cytometry analyses showed the proportion of O4+ cells of the four MSA patient-derived iPSC lines **(G)** Terminal differentiation led to the generation of MBP+ oligodendrocyte cells. **(H, I)** The four MSA patient-derived iPSC were also able to differentiate into neural and other glial lineages, GFAP+ astrocytes in **(H)** and MAP2+ neurons in **(I)** were obtained. Nuclei were counterstained with DAPI in **(D-I)**. Scale bars **(B)** 1000 µm; **(C)** 400 µm; **(D, G)** 20 µm; **(E)** 200 µm; and **(H, I)** 50 µm.

OLs have been identified as the cell type primarily affected in MSA disease. Their cytoplasm accumulates aggregates of pathological fibrillary forms of aSyn in GCIs that propagate throughout the brain as the disease progresses (44). To validate the use of MSA hiPSCs for disease modeling and drug screening, they were differentiated to OLs following the protocol from Douvaras & Fossati 2015 (24). This protocol recapitulates spinal cord embryonic development and consists of four main stages (**Figure 4A**): (1) the specification of the pMN domain in monolayer culture via dual-SMAD inhibition and all-*trans* RA and sonic hedgehog (SHH) signaling; (2) the enrichment of the pMN progenitors (OLIG2+ cells) by generating cell aggregates in suspension; (3) the differentiation to OPCs (O4+ cells) by treating the plated cell aggregates with key factors as PDGF, NT3, T3, IGF-1 and HGF; and (4) the terminal maturation to OLs (O4+MBP+ cells) by the withdrawal of the previously mentioned factors. Due to budget constraints, we were only able to differentiate four MSA hiPSC lines: MSA01, MSA03, MSA04, and MSA06. We excluded MSA02 because this case presented the most extensive and severe co-pathologies, including high-grade chronic traumatic encephalopathy (CTE) related to repeated head trauma, Alzheimer’s disease with an intermediate Amyloid-Braak-CERAD (ABC) score, and cerebral small vessel disease (SVD). Also, MSA05 was not included as no brain donation was available for this case.

The OL differentiation status was monitored at various timepoints (**Figures 4B, C**; **Supplementary Figure 4A**). First, we had to adjust the initial plating density of each patient-derived iPSC line to obtain an overconfluent culture at day 12, characterized by cells piling up and forming 3D structures. At day 30, we could observe that the cell clusters in suspension were homogeneous and characterized by being round (spheres), brown with a darker core, and 300 - 800 µm in diameter (**Figure 4B**). When these spheres were plated into laminin/poly-L-ornithine coated wells, cells started to migrate out. Around day 45, we could confirm the presence of neurons, clearly distinguishable by their long processes that extend out from the cell soma (**Figure 4C**). From day 55 and onwards, we detected the emergence of many highly branched cells, which were positive for the oligodendrocyte marker O4 (**Figures 4D, E**). All the MSA hiPSC lines differentiated efficiently to OPCs, as evidenced by the flow cytometry results at day 75 showing O4+ cell percentages ranging from 62% to 81% (**Figure 4F**; **Supplementary Figure 4B**). The terminal differentiation of OPCs to more mature OLs from day 75 to day 90 was demonstrated by the presence of cells co-expressing O4 and myelin basic protein, MBP (**Figure 4G**) (representative image of one MSA hiPSC line). We found glial fibrillary acidic protein (GFAP, astroglial marker) (**Figure 4H**) and microtubule-associated protein 2 (MAP2, neuronal marker) (**Figure 4I**) expressing cells in our *in vitro* culture (representative image of one MSA hiPSC line). These immunofluorescence images evidenced that the protocol also generated astrocytes and neurons (**Supplementary Figure 4C**), which are essential for supporting the survival and growth of oligodendrocytes.

## 4 Discussion

Here, we describe the generation and characterization according to ISSCR standards of six iPSC lines from MSA patients. For each iPSC line, we created a set of three clones; however, in this manuscript, we only show the results of one clone. MSA is an oligodendroglial α-synucleinopathy with two predominant clinical variants: MSA-P (with parkinsonian symptoms) and MSA-C (with cerebellar symptoms). Significantly, we included both MSA-P and MSA-C cases in our iPSC collection to *in vitro* recapitulate disease pathobiology and heterogeneity. Next to their clinical diagnosis, five of the six cases were autopsy-confirmed for MSA, avoiding the common hurdle of potential misdiagnosis that has been described to be up to 20% of cases (45) and, thus, providing an accurate and robust iPSC disease model.

Thanks to the collaboration with the CMSAR, we received human adult dermal fibroblasts (HDF-ad) from MSA patients, which were reprogrammed into iPSCs using SeV. Access to targeted neural or glial cell types is restricted to postmortem brain samples after many years of disease evolution. Hence, iPSCs emerge as a valuable tool to study MSA since they can generate an unlimited supply of disease-specific cell types, such as OLs. HDF-ad were chosen as the donor cell source in the reprogramming process for multiple reasons. They are easily accessible with a skin biopsy punch. At the present time, the majority of iPSCs obtained to study MSA are derived from fibroblasts (16, 17). MSA is considered multifactorial, caused by the combination of both genetic and environmental factors, and HDF-ad retain the genetic risk variants that may be associated with the development of late-onset neurodegenerative diseases (46). Recently, genome-wide association studies (GWAS) from 888 MSA patients identified four novel risk loci (*GAB1*, *lnc-LRRC49-3*, *TENM2*, and *RABGEF1*) and three novel susceptibility genes within these loci (*USP38-DT*, *KCTD7*, and *lnc-KCTD7-2* (47). The reprogramming and long-term passaging of the adult fibroblasts-derived iPSCs progressively erase the epigenetic signature characteristic of their somatic tissue of origin (48, 49). Current research is focusing on reintroducing the pathological epigenetic footprint of neural and glial cell types. For instance, mimicking ageing hallmarks have been explored through methods such as exposing the cells to replicative stress or to pharmacological treatments like hydrogen peroxide (50).

The effectiveness of iPSC-based disease modeling for identifying the molecular mechanisms underlying pathology and for discovering novel therapies depends on the use of consistently high-quality cells. We followed a detailed quality control protocol to verify iPSC cell identity, genetic stability, pluripotent nature, and differentiation potential (27, 51). First, the authentication of the newly generated iPSC lines was achieved by comparing their short tandem repeat (STR) profile with the parental patient’s fibroblasts. Also, a combination of karyotyping and copy number variation (CNV) analyses were performed to examine whether genetic changes occurred during the reprogramming and the prolonged passage of the cells. These two techniques provide complementary information. G-banding karyotyping captures a genome-wide snapshot of the cells, facilitating an unbiased analysis of any structural rearrangement larger than approximately 5–10 megabases (28). CNV determination with digital PCR allows identifying specific gains or deletions of portions of chromosomes with a resolution limited to >10 bp-long (21). The analysis of hundreds of human pluripotent stem cells (hPSCs) from laboratories worldwide allowed the identification of recurrent gains that generate a selective advantage to their capacity of self-renewal and/or maintenance of the pluripotent state, becoming fixed over time (35, 52–54). For instance, the 20q11.21 amplification causes the increased expression of the *BCL2L1* gene, which drives an anti-apoptotic role and has a negative effect on the neuroectodermal differentiation capacity through the TGBβ signaling (30, 31, 52, 55). For hPSCs, the screening of six hotspots covers 62.2% of the reported abnormalities (21). The G-banding karyotyping and the digital PCR CNV analysis were performed after reprogramming and passaging the cells to efficiently remove the Sendai vectors. However, these genomic abnormalities can be acquired at any timepoint during the *in vitro* iPSC culture, so both techniques should be done routinely, for instance every 12 weeks (28).

We demonstrated the commitment of the six MSA hiPSC lines into the three embryonic germ layers. The current gold-standard method is based on the commercial STEMdiff™ trilineage differentiation kit (STEMCELL Technologies), because it is rapid (5–7 days) and reproducible. We also decided to perform the EB-based trilineage differentiation because it generates more developmentally mature tissues with a distinguishable morphogenesis. Furthermore, MSA hiPSCs could be differentiated into OPCs and OLs, offering a valuable platform for *in vitro* disease modeling. We followed the Douvaras and Fossati 2015 protocol, which was designed to recapitulate the spinal cord embryonic development using a defined growth factor (GF)-rich medium (24). It gave us an efficient outcome, with around 60 - 80% of O4+ expressing cells. However, this protocol requires long culture periods (75–90 days), which is time- and money-consuming. To overcome these limitations, other OL differentiation protocols based on the forced expression of transcription factors (TFs) have been published. Traditionally, TFs delivery has been viral vector-mediated, introducing the potential for off-target effects like insertional and recombinational mutagenesis (56, 57). This could bring confounding bias in our *in vitro* disease model. Recently, a synthetic modified messenger RNA (smRNA)-based method has been developed, encoding a modified form of *OLIG2* (58). All present OL differentiation protocols generate mixed glial cultures, since astrocytes and/or neurons are essential to *in vitro* support OL survival and growth (24). Therefore, a purification step is necessary to isolate the OPC and OLs. Based on our experience, the dissociation of the culture as single cells to be able to sort the O4+ cells via fluorescence-activated cell sorting (FACS) or via magnetic-activated cell sorting (MACS) caused some cell death.

For rare diseases as MSA, it is crucial to establish hiPSC banks to encourage research and facilitate collaboration between scientists and clinicians worldwide. They should rely on multiple cell lines to accurately represent the potential sources of disease variability, including the clinical manifestations and patient systemic factors (genetic background, age, sex, presence of comorbidities, etc.). For instance, in our cell collection, patients have different predominant motor and autonomic symptoms, the age of onset range from 49 to 71 years, and the survival rate is 7 to 15 years from diagnosis. Four out of five cases had copathology in the form of amyloid, tau or small vessel disease. Late-onset neurodegenerative diseases are characterized by the accumulation of multiple neuropathologies, being influenced in part by patient age and disease duration and resulting in heterogeneous combinations (59, 60). Significantly, we could inform about all the comorbidities present in each patient, since they could have an impact on disease progression and treatment response.

In conclusion, we successfully generated and comprehensively characterized six iPSC lines derived from MSA patient-derived fibroblasts. Given the inherent limitations of postmortem tissue studies, MSA patient-derived fibroblasts provide a valuable and accessible resource for studying the genetic and molecular mechanisms underlying MSA disease. By including both pathologically confirmed MSA-P and MSA-C cases, we aimed to recapitulate disease heterogeneity *in vitro*. The iPSC lines underwent rigorous quality control, including STR profiling, G-banded karyotyping, CNV hotspot screening, and pluripotency validation, ensuring genomic stability and suitability for downstream applications. Using a defined, GF-based protocol, we successfully differentiated these lines into O4+ oligodendrocyte progenitor cells and O4+MBP+ mature oligodendrocytes. Despite the time-consuming nature of this protocol, it yielded high differentiation efficiency without the off-target effects associated with viral vector-mediated TF overexpression. This platform, anchored in clinically and pathologically well-characterized cases, offers technical robustness and translational relevance for studying α-synuclein pathology in a human model. By leveraging patient-specific lines, we provide a valuable tool for understanding MSA pathogenesis, enabling therapeutic target identification, drug screening, and laying the groundwork for future individualized medicine approaches.

## Data Availability

The original contributions presented in the study are included in the article and **Supplementary Material**. Further inquiries can be directed to the corresponding authors.

## Supplementary material

Supplementary Figure 1 Evaluation of the ectodermal differentiation potential based on the STEMdiff™ Trilineage Differentiation Kit. The 3-channel immunofluorescence imaging with PAX6, NES and DAPI for MSA02, MSA03, MSA04, MSA05 and MSA06 patient-derived iPSC lines. Scale bars, 20 µm.

Supplementary Figure 2 Evaluation of the endodermal differentiation potential based on the STEMdiff™ Trilineage Differentiation Kit. The 3-channel immunofluorescence imaging with SOX17, FOXA2 and DAPI for MSA02, MSA03, MSA04, MSA05 and MSA06 patient-derived iPSC lines. Scale bars, 20 µm.

Supplementary Figure 3 Evaluation of the mesodermal differentiation potential based on the STEMdiff™ Trilineage Differentiation Kit. The 3-channel immunofluorescence imaging with TBXT, NCAM1 and DAPI for MSA02, MSA03, MSA04, MSA05 and MSA06 patient-derived iPSC lines. Scale bars, 20 µm.

Supplementary Figure 4 Generation of oligodendrocyte progenitor cells and oligodendrocyte cells from four MSA patient-derived iPSC lines, related to **Figure 4**. **(A)** Distinctive moments of the oligodendrocyte differentiation protocol: at day 0, hiPSC colonies had a diameter around 100 – 250 µm; at day 12, the culture was overconfluent; and at day 55, branched cells growing around the neuronal-like cells were visible. **(B)** At day 75, a negative control for flow cytometry was obtained by staining the differentiated cells with the fluorescently-labeled secondary antibody alone; and so the gating strategy for O4+ quantification was provided. **(C)** Two representative images demonstrating that the differentiation protocol generated MBP+ oligodendrocytes, growing together with supporting GFAP+ astrocytes and MAP2+ neurons. Scale bars **(A)** 400 µm; and **(C)** 50 µm.
