# Supplementary figures and images for: Generation and characterization of human induced pluripotent stem cells from neuropathologically confirmed multiple system atrophy patient-derived fibroblasts
Source: Front Immunol. 2026 Feb 23;17:1641981. doi: 10.3389/fimmu.2026.1641981 (PMC12967983; doi:10.3389/fimmu.2026.1641981)

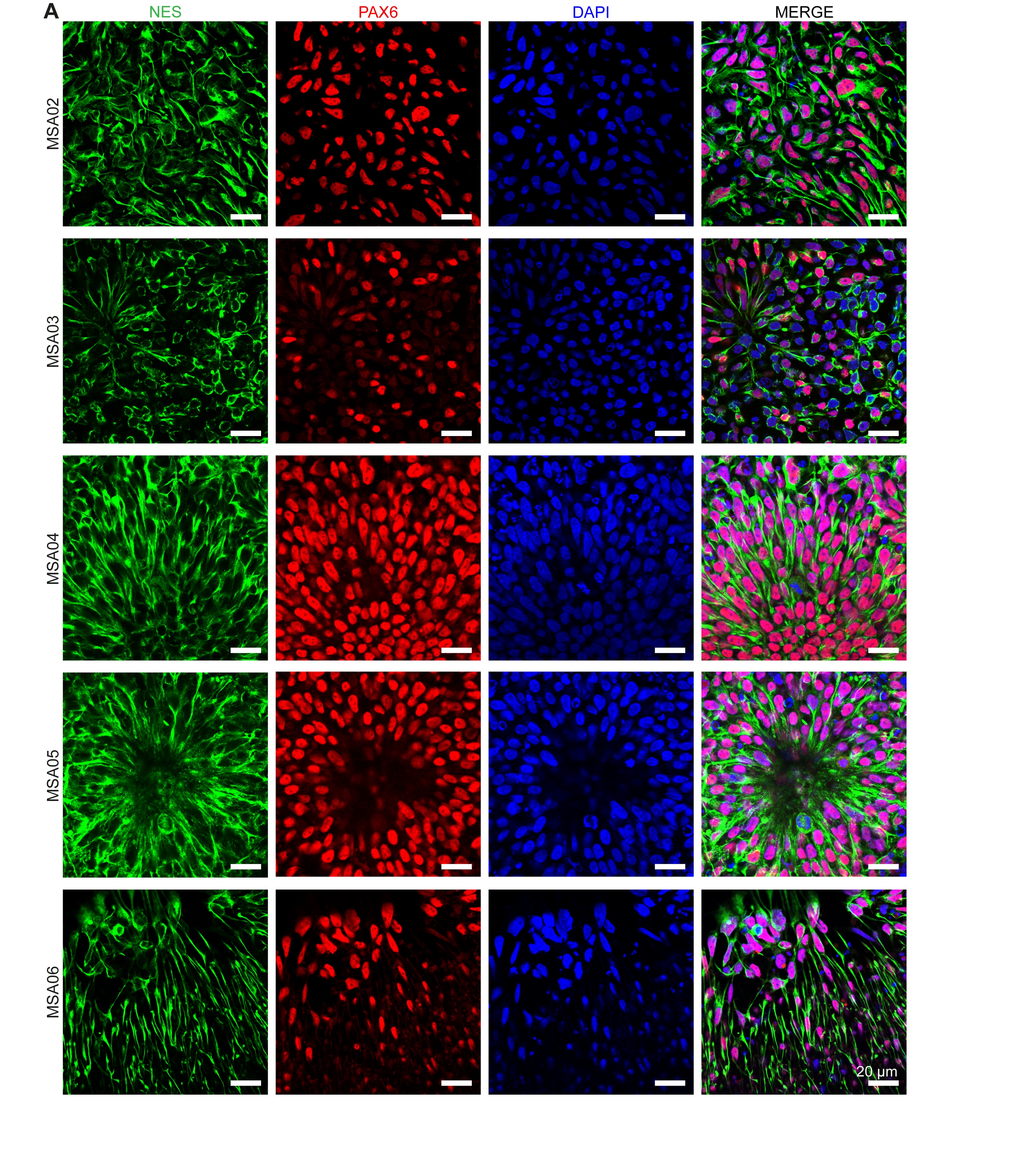

Supplement: Supplementary Figure 1 — Evaluation of the ectodermal differentiation potential based on the STEMdiff™ Trilineage Differentiation Kit. The 3-channel immunofluorescence imaging with PAX6, NES and DAPI for MSA02, MSA03, MSA04, MSA05 and MSA06 patient-derived iPSC lines. Scale bars, 20 µm. [file Image1.jpeg]

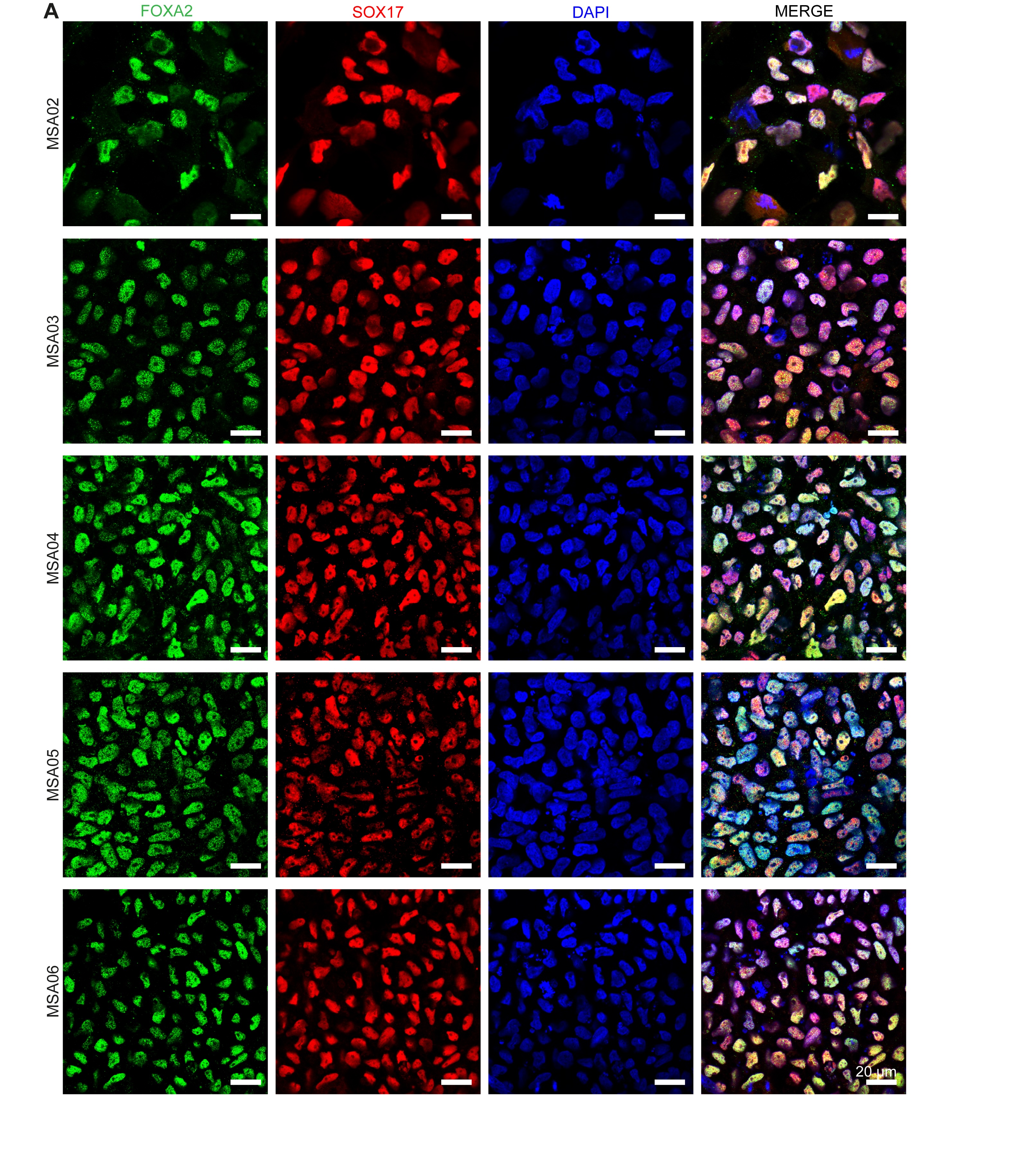

Supplement: Supplementary Figure 2 — Evaluation of the endodermal differentiation potential based on the STEMdiff™ Trilineage Differentiation Kit. The 3-channel immunofluorescence imaging with SOX17, FOXA2 and DAPI for MSA02, MSA03, MSA04, MSA05 and MSA06 patient-derived iPSC lines. Scale bars, 20 µm. [file Image2.jpeg]

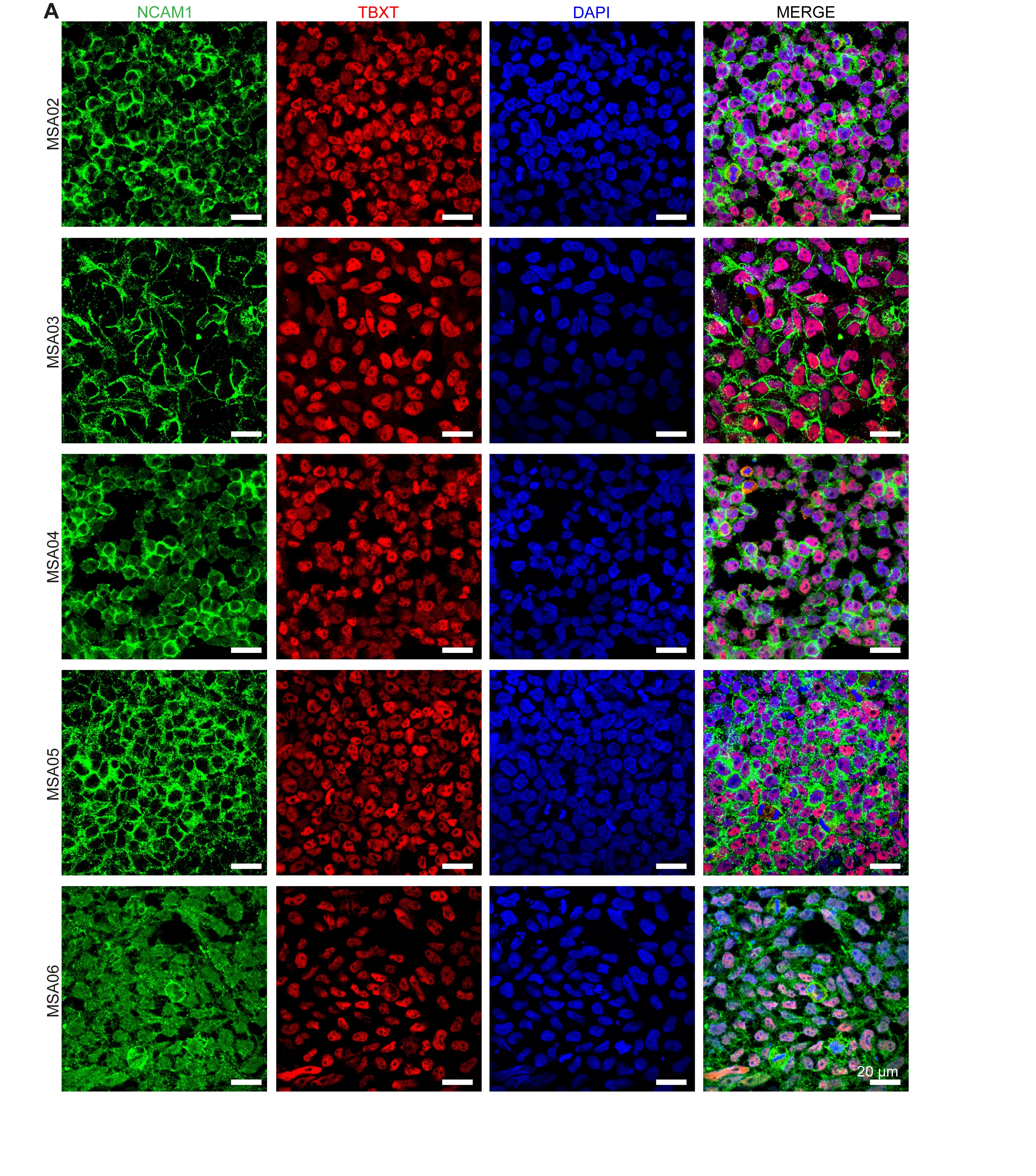

Supplement: Supplementary Figure 3 — Evaluation of the mesodermal differentiation potential based on the STEMdiff™ Trilineage Differentiation Kit. The 3-channel immunofluorescence imaging with TBXT, NCAM1 and DAPI for MSA02, MSA03, MSA04, MSA05 and MSA06 patient-derived iPSC lines. Scale bars, 20 µm. [file Image3.jpeg]

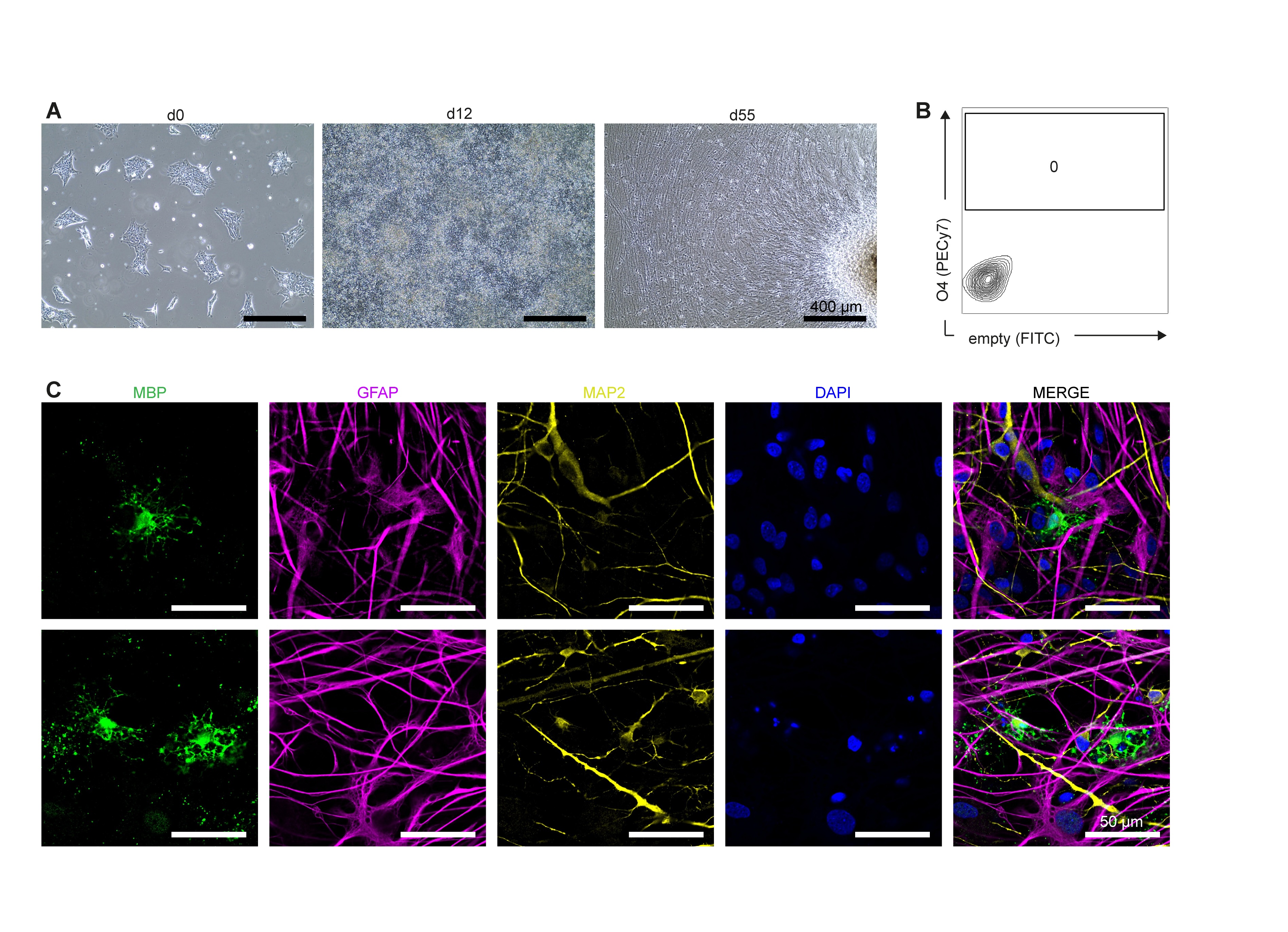

Supplement: Supplementary Figure 4 — Generation of oligodendrocyte progenitor cells and oligodendrocyte cells from four MSA patient-derived iPSC lines, related to Figure 4. (A) Distinctive moments of the oligodendrocyte differentiation protocol: at day 0, hiPSC colonies had a diameter around 100 – 250 µm; at day 12, the culture was overconfluent; and at day 55, branched cells growing around the neuronal-like cells were visible. (B) At day 75, a negative control for flow cytometry was obtained by staining the differentiated cells with the fluorescently-labeled secondary antibody alone; and so the gating strategy for O4+ quantification was provided. (C) Two representative images demonstrating that the differentiation protocol generated MBP+ oligodendrocytes, growing together with supporting GFAP+ astrocytes and MAP2+ neurons. Scale bars (A) 400 µm; and (C) 50 µm. [file Image4.jpeg]
